# Agent-based representations of objects and actions in the monkey pre-supplementary motor area

**DOI:** 10.1073/pnas.1810890116

**Published:** 2019-01-29

**Authors:** Alessandro Livi, Marco Lanzilotto, Monica Maranesi, Leonardo Fogassi, Giacomo Rizzolatti, Luca Bonini

**Affiliations:** ^a^Department of Medicine and Surgery, Neuroscience Unit, University of Parma, 43125 Parma, Italy;; ^b^Parma Unit, Institute of Neuroscience of Centro Nazionale delle Ricerche (CNR), 43125 Parma, Italy

**Keywords:** mirror neuron, macaque, object processing, action prediction, pre-SMA

## Abstract

Information about objects around us is essential for planning actions and for predicting those of others. Here, we studied pre-supplementary motor area F6 neurons with a task in which monkeys viewed and grasped (or refrained from grasping) objects, and then observed a human doing the same task. We found “action-related neurons” encoding selectively monkey’s own action [self-type (ST)], another agent’s action [other-type (OT)], or both [self- and other-type (SOT)]. Interestingly, we found “object-related neurons” exhibiting the same type of selectivity before action onset: Indeed, distinct sets of neurons discharged when visually presented objects were targeted by the monkey’s own action (ST), another agent’s action (OT), or both (SOT). Notably, object-related neurons appear to signal self and other’s intention to grasp and the most likely grip type that will be performed, whereas action-related neurons encode a general goal attainment signal devoid of any specificity for the observed grip type. Time-resolved cross-modal population decoding revealed that F6 neurons first integrate information about object and context to generate an agent-shared signal specifying whether and how the object will be grasped, which progressively turns into a broader agent-based goal attainment signal during action unfolding. Importantly, shared representation of objects critically depends upon their location in the observer’s peripersonal space, suggesting an “object-mirroring” mechanism through which observers could accurately predict others’ impending action by recruiting the same motor representation they would activate if they were to act upon the same object in the same context.

## Significance

Social animals exploit information about objects for planning actions and for predicting those of others. Here, we show that pre-supplementary motor area F6 hosts different types of neurons responding to visually presented objects when they are targeted by the monkey’s own action (self-type), another agent’s action (other-type), or both (self- and other-type). These findings suggest the existence in area F6 of an “object-mirroring” mechanism, which allows observers to predict others’ impending action by recruiting the same motor representation they would use for planning their own action in the same context, before the activation of classical “action-mirroring” mechanisms.

Several regions in the primate brain host neurons that encode both one’s own and others’ actions (1–3). These neurons, called “mirror neurons,” endow primates with a putatively agent-invariant code for actions (4), which is deemed to be at the basis of several cognitive functions and social interaction skills (5, 6).

Despite this shared sensorimotor code, subjects typically do not confound self with others. This may be achieved not only by means of sensory signals coming from the body, which are exclusively correlated with own actions, but also thanks to single neurons selectively encoding others’ observed action (without any motor component), which have been found in various brain regions (7–9) and especially in the pre-supplementary motor area F6 (10). In this latter region, Yoshida et al. (10) tested neurons with a role reversal arm-reaching task in which two monkeys alternatively played the role of actor and partner. They found three main types of neurons: “partner-type” neurons, encoding selectively others’ action; “self-type” neurons, encoding one’s own action; and “mirror-type” neurons, encoding both one’s own and others’ action. These findings demonstrated a complex agent-based representation of reaching actions in the mesial frontal cortex, which may be critical during interaction with others. Indeed, the pre-supplementary motor cortex has been recently identified as a key node of a brain network dedicated to processing observed social interactions (11).

During social interaction, it is extremely useful to predict the most likely action another may perform rather than simply reacting to it after its onset. To this purpose, the capacity to exploit contextual information about potential target objects is of critical importance for animals living in complex social groups, like primates. Nonetheless, its possible neural basis remains largely unknown. Anticipatory signals at the single-neuron level (12–15) have been identified during the visual presentation of a potential target, before any observable movement. However, these signals have generally been considered an unspecific, anticipatory activation of the impending motor action rather than a specific representation of the observed object. A notable exception is constituted by a few human studies that have suggested the existence of a rough, agent-invariant representation of objects in terms of their motor affordance when they are targeted both by one’s own and another agent’s action (16, 17). Nonetheless, the neuronal mechanisms for agent-based motor representation of real solid objects and, most importantly, their possible link with self- and other-action coding, remain unknown.

To address these issues, in the present study, we recorded single-neuron activity from area F6 of two monkeys while they performed a visuomotor Go/No-Go reaching–grasping task (18) and while they observed the same task performed by an experimenter in the same context. In agreement with previous studies (10), we found distinct sets of action-related neurons encoding selectively monkey’s own reaching–grasping action, other’s action, or both. Most interestingly, we found that even neurons responding during the visual presentation of graspable objects (object-related neurons) showed agent selectivity, discharging when a visually presented object was targeted by the monkey’s own action (self-type neurons), another agent’s action (other-type neurons), or both (self- and other-type neurons). By single-neuron analysis and population decoding approaches, we provide evidence for the existence of an “object-mirroring” mechanism that allows subjects to recruit the very same motor representation of an object they would use for planning their own action to precisely predict whether (Go/No-Go) and how (grip type) another agent will act when facing the same object in the same context.

## Results

**Fig. 1.**
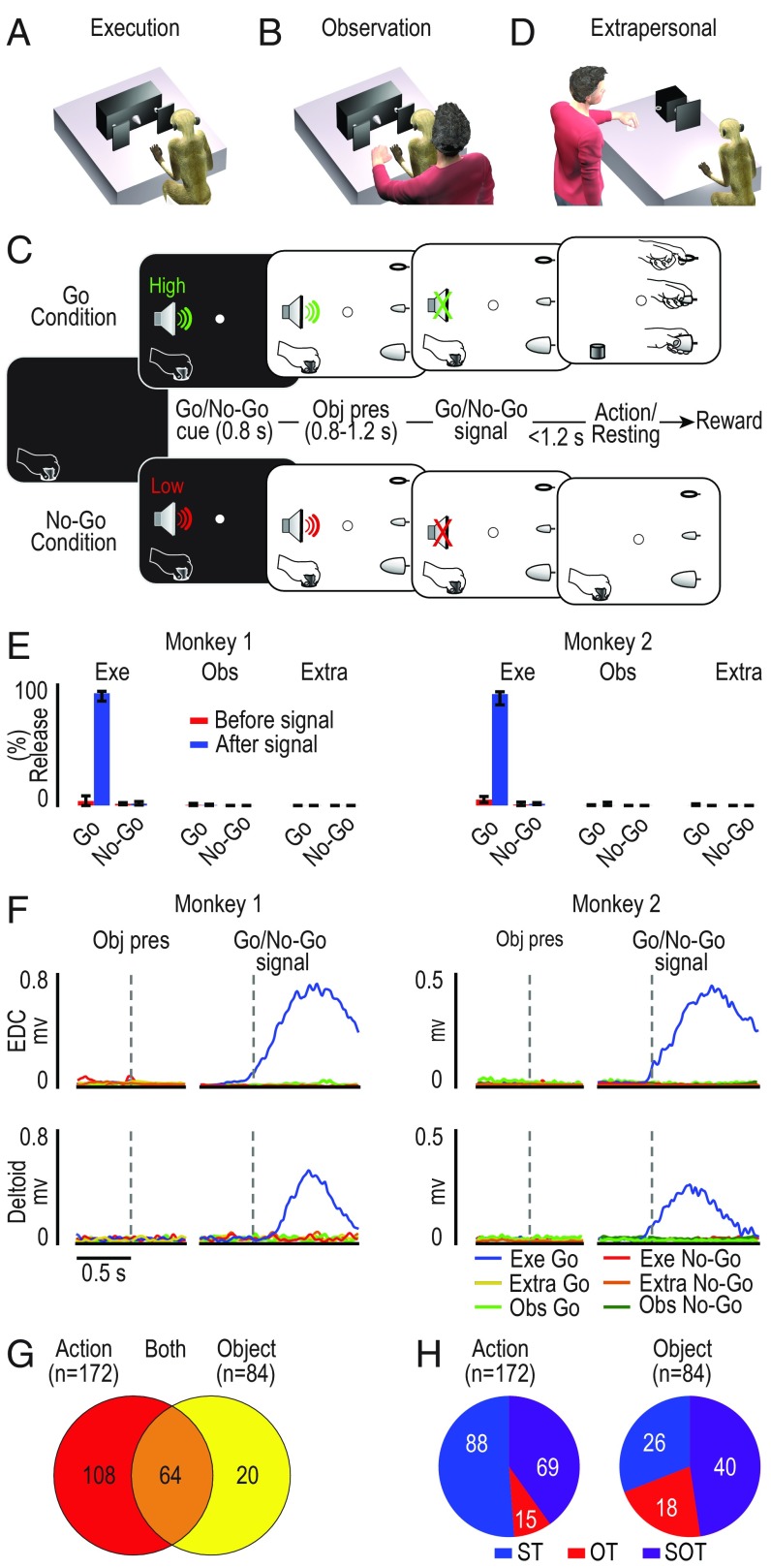
Behavioral paradigm, behavioral data, and main neuronal categories. (*A* and *B*) Behavioral setup for the execution (*A*) and observation (*B*) task. (*C*) Temporal sequence of task events. Each trial started when the monkey, with its hand in the starting position, engaged fixation in complete darkness. A high (Go cue) or low (No-Go cue) tone was presented and remained on during the subsequent object presentation phase (Obj pres). When the sound stopped (Go/No-Go signal), the agent (monkey or experimenter) had to reach, grasp, and pull the target (Go trial) or to remain still (No-Go trial), maintaining fixation for the entire duration of the trial (*Methods*). (*D*) Behavioral setup for the extrapersonal observation task. A smaller rotating device allowed to present the same three objects used in the basic execution and observation tasks. (*E*) Median percentage (M1, *n* = 5; M2, *n* = 9 sessions) of events in which the monkey released the manipulandum in the period before (red) and after (blue) the Go and No-Go signal in the execution (Exe), observation (Obs), and extrapersonal (Extra) tasks. Error bars indicate the 5th and 95th percentiles. (*F*) EMG temporal activation profile of the distal [extensor digitorum communis (EDC)] and proximal (deltoid) muscles recorded during different task conditions aligned (dashed lines) on object presentation (Obj pres) and, after the gap, on the Go/No-Go signal. (*G*) Number of task-related neurons classified as action related, object related, or both. (*H*) Agent selectivity of action- and object-related neurons (OT, other-type neurons; SOT, self- and other-type neurons; ST, self-type neurons).

Two monkeys (M1 and M2) were trained to perform (Fig. 1*A*) or observe (Fig. 1*B*) a visuomotor Go/No-Go task with three possible target objects (a ring, a small cone, and a big cone), each affording a different type of grip (hook grip, precision grip, and power grip) (*SI Appendix*, Fig. S1). In the execution task (Fig. 1*A*), monkeys were instructed by an auditory Go/No-Go cue either to reach, grasp, and pull the visually presented object (Go condition), or to refrain from grasping it (No-Go condition) (Fig. 1*C*). In the observation task, ran in a separate block of trials, the experimenter was standing behind the monkey (Fig. 1*B*), which observed the sequence of events while remaining still (in both Go and No-Go condition) and maintaining fixation. Thus, the Go trials of the observation task were identical to those of the execution task from the monkey’s point of view (same objects, in the same position, preceded by the same auditory cue), but the presence of the experimenter’s hand cued the monkey to refrain from moving because in that context the Go cue was addressed to the experimenter. Neuronal activity was also recorded during a modified version of the observation task in which the experimenter performed all task conditions on a device located in the monkey’s extrapersonal space, thus with the target object completely outside of the monkey’s reach (extrapersonal task, Fig. 1*D*).

### Monkeys React Differently to the Same Cue Depending on Task Context.

Both animals, depending on the task context, responded differently to the auditory Go cue (Fig. 1*E*). They typically reacted to the end of the Go auditory cue (Go-signal) by detaching the hand from the starting position and reaching for the target in the execution task (M1, 91.8% of the trials; M2, 91.5%) but not in the observation task (<5% of the trials in both monkeys). Furthermore, during the observation tasks, the location of the target within the peripersonal or extrapersonal space, and hence the monkey’s possibility to interact with it, did not affect the probability (<1% of the trials in each observation task and in both monkeys; Fig. 1*E*) of incorrectly detaching the hand from the starting position.

The analysis of errors suggests that monkeys discriminated the behavioral meaning of the auditory cues depending on task context. However, they could exhibit a subtler preparatory activity that does not result into an overt behavioral error but could nonetheless affect muscles activity. To resolve this question, we recorded electromyographic (EMG) activity by placing surface EMG electrodes over a proximal and a distal muscle of the monkeys’ forelimb during all task conditions, after the end of the neuronal recording sessions. We only observed significant muscle activation (Bonferroni post hoc tests, *P* < 10^−10^ for all comparisons) following the Go-signal in the execution task (Fig. 1*F*, blue lines). Altogether, these findings indicate that both monkeys discriminate whether the cues were addressed to themselves or to the experimenter, depending on the task context.

### Agent-Based Representation of Manual Actions and Graspable Objects at the Single-Neuron Level.

We recorded the activity of 306 single neurons during the execution and observation task, out of which 192 were classified as task related because they encoded executed and/or observed action, object, or both (*Methods*). Single-neuron activity was sampled from the same chronic multielectrode probes used in a previous study focused on the motor properties of area F6 (18). In that study, we provided extensive histological and functional (intracortical microstimulation and single-neuron visuomotor properties) evidence that the investigated area is completely within area F6, encompassing most of its rostrocaudal extension (*SI Appendix*, Fig. S2).

One set of neurons responded only during execution and/or observation of the action (*n* = 108), a second set responded only to the visual presentation of the object during the execution and/or the observation task (*n* = 20), and a third set (*n* = 64) encoded both object presentation and action execution/observation (Fig. 1*G*). The three sets of neurons were substantially intermingled along the rostrocaudal extent of the recorded regions in both monkeys (*SI Appendix*, Fig. S2). All of the neurons responding at least during action execution and/or observation were classified as action related (*n* = 172), and all of those discharging at least to object presentation were classified as object related (*n* = 84). Based on this classification, we first asked whether and to what extent F6 neuronal representations of object and action differed depending on the agent (i.e., monkey or experimenter; *Methods*): We found evidence of agent selectivity in both (Fig. 1*H*).

### Action-Related Neurons: Agent-Based Representation of Reaching–Grasping Actions.

**Fig. 2.**
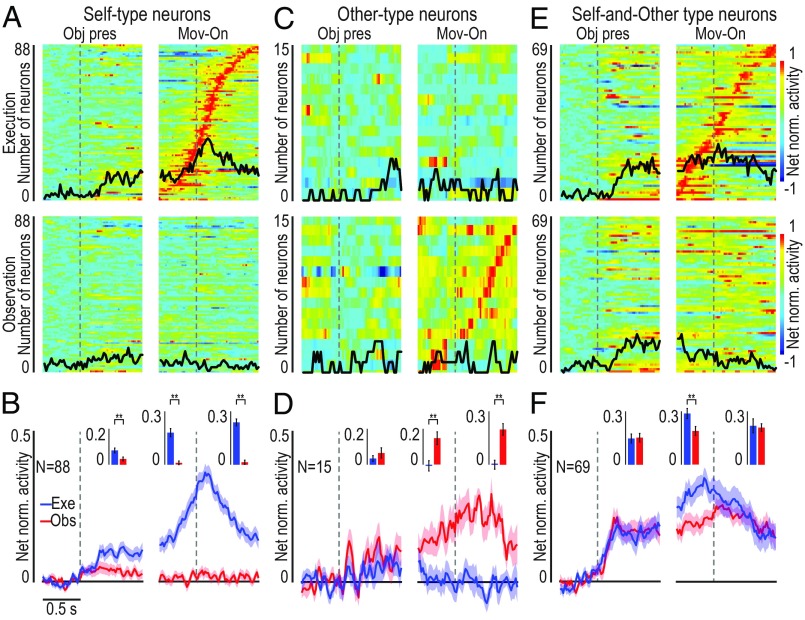
Single-neuron and population activity of ST, OT, and SOT action-related neurons. (*A*, *C*, and *E*) Heat map of single-unit net normalized activity for ST (*A*), OT (*C*), and SOT (*E*) action-related neurons. Each set of neurons is aligned to the object presentation (vertical dashed line in the *Left*), and then (after the gap) to the movement onset (i.e., detachment of the hand from the starting position) during the execution (*Top* row) and observation (*Bottom* row) task. Neurons are ordered based on the timing of their peak of activity in the movement period of the execution (*A* and *E*) or observation (*C*) task, with the earliest on *Bottom* (*SI Appendix*, Fig. S3). The response to the three objects is averaged. The black line superimposed to each plot indicates the number of neurons (same scale on the *Left*) showing selectivity for the type of object (sliding ANOVA, *P* < 0.05, uncorrected). (*B*, *D*, and *F*) Time course of the mean net normalized population activity for the same neurons shown in the correspondent panel above during the execution (blue) and observation (red) task. The shading around each line indicates 1 SE. Histograms in the *Insets* of each panel show the mean activity in each epoch for the two conditions. Paired-samples *t* tests: *P < 0.05, **P < 0.01.

Our reaching–grasping task allowed us to distinguish three main activation patterns of F6 action-related neuron (Fig. 1*H*), which we defined by adopting the same classification criteria proposed by a previous study on arm-reaching actions (10). The majority of action-related neurons (*n* = 88; 51%) became active only during monkey’s own action [self-type (ST) neurons; Fig. 2 *A* and *B*], some (*n* = 15, 9%) only during experimenter’s action [other-type (OT) neurons; Fig. 2 *C* and *D*], and another set (*n* = 69, 40%) during both monkey’s and experimenter’s action [self- and other-type (SOT) neurons; Fig. 2 *E* and *F*]. These latter neurons exhibited the same type of response pattern that characterizes classical mirror neurons, originally recorded from the monkey ventral premotor cortex (7, 19) and deemed to provide a shared neural substrate for the representation of self and others’ action.

**Fig. 3.**
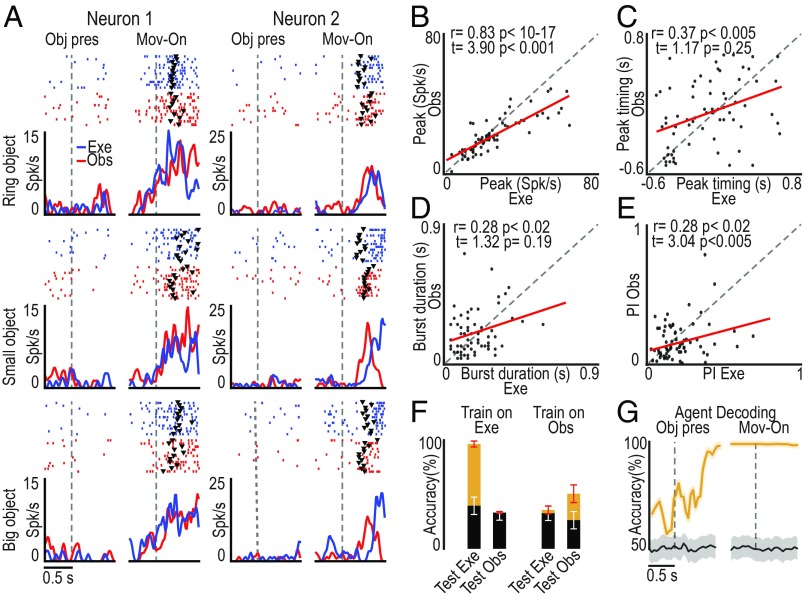
Relationship between the response of SOT action-related neurons (*n* = 69) during own-action execution and other’s action observation. (*A*) Single-neuron response (raster and line plot) during task execution (Exe, blue) and observation (Obs, red) with the three target objects. Alignments are as in Fig. 2. Black triangular markers indicate object-pulling onset. (*B*–*E*) Correlation plots of neurons’ peak of activity (*B*), peak timing (*C*), burst duration (*Methods*) (*D*), and object preference index (PI) (*E*) (*Methods* and *SI Appendix*, Fig. S4*A*) between action execution and observation (−0.5/+0.8-s interval relative to movement onset). Paired-sample *t* tests were performed to compare the values of each parameter between the tasks. (*F*) Object classification accuracy during execution and observation trials based on training a classifier with data collected during either one of the two tasks (orange, real data; black, shuffled data). Red and white error bars indicate 1 SD for real and shuffled data, respectively. (*G*) Time course of agent decoding accuracy during task execution and observation (orange line). Black line, chance level (*Methods*). Shaded area around the curves: ±1 SD over resample runs.

Examples of two SOT action-related neurons are shown in Fig. 3*A*: The monkey actively grasped the target object in the execution task, whereas it remained still during the observation task, but these neurons encoded both self- and other-action, although with some differences between the two conditions (e.g., neuron 2). To quantitatively assess the relationship between self- and other-action coding, we statistically analyzed parameters of action-related neuronal activity in the two contexts (execution and observation). We found that the peaks of activity (Fig. 3*B*) and their timing (Fig. 3*C*), the bursts’ duration (Fig. 3*D*), and the neural preference for objects (Fig. 3*E*) were significantly correlated between the two tasks, although both the peak of activity and the neural preference for objects were greater during the execution relative to the observation task (Fig. 3 *D* and *E* and *SI Appendix*, Fig. S4).

To further clarify whether and to what extent SOT action-related neurons provide a shared code for self- and other-action, we performed a cross-modal decoding analysis (see *Methods*) aimed at discriminating the target objects during both the execution and the observation task using a classifier (20) trained on the activity recorded during either action execution (Fig. 3*F*, Train on Exe) or action observation (Fig. 3*F*, Train on Obs). The results did not show evidence of cross-modal decoding: Indeed, object decoding accuracy was above chance level only when training and testing data for the decoder came from task execution, suggesting that this effect is mostly due to a somatomotor signal related to the grip type rather than to a visuomotor signal related to object features. This finding indicates that SOT neurons, despite their activation during both action execution and observation, convey more detailed information about one’s own than others’ action and can therefore contribute to self–other distinction. To more directly assess this hypothesis, we trained the classifier to distinguish self- from other-action (regardless of the target) based on the activity of SOT neurons recorded during own-action execution and other-action observation: by testing the decoding performance (*Methods*), we found that SOT neurons’ activity makes it possible to decode the agent (monkey or experimenter) with high accuracy even before movement onset (Fig. 3*G*), likely because of the self-bias that is also evident in SOT action-related neurons’ population activity (Fig. 2 *E* and *F*).

### Object-Related Neurons: Agent-Based Representation of Graspable Objects.

**Fig. 4.**
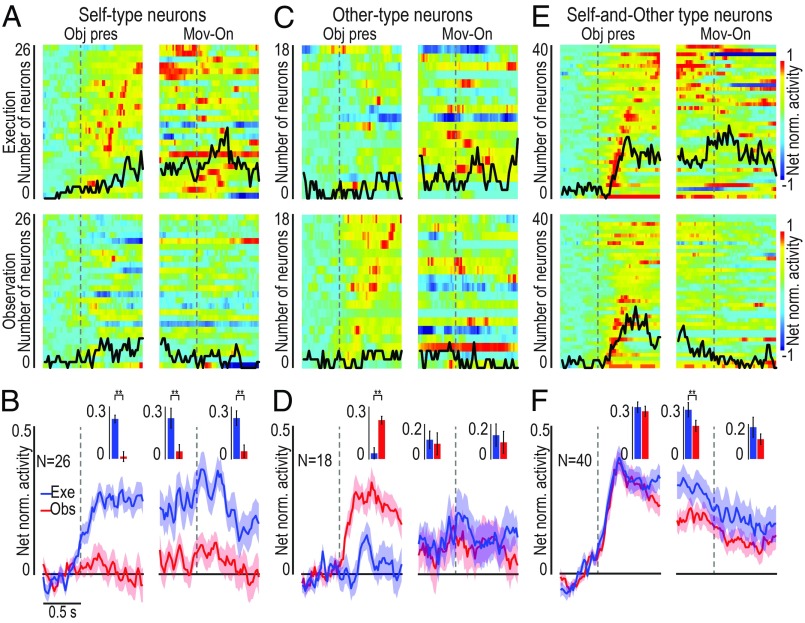
Single-neuron and population activity of self-type (ST), other-type (OT), and self- and other-type (SOT) object-related neurons. (*A*, *C*, and *E*) Heat map plots of the temporal activation profile of single-unit net normalized activity for ST (*A*), OT (*C*), and SOT (*E*) object-related neurons. Neurons are ordered based on the timing of their peak of activity in the object presentation period of the execution task, in case of ST and SOT neurons (*A* and *E*), or of the observation task, in the case of OT neurons (*C*), with the earliest on the *Bottom* for all plots. (*B*, *D*, and *F*) Time course and intensity of the mean net normalized population activity for the three sets of neurons shown in the correspondent panels above, during the execution (blue) and observation (red) task. All conventions are as in Fig. 2. Pired-samples *t* tests: **P < 0.01.

The target presentation response of object-related neurons could show agent selectivity as well (Fig. 1*G*). Some object-related neurons (*n* = 26, 31%) became active only when the visually presented object was the target of monkey’s own action (ST neurons; Fig. 4 *A* and *B*), 18 (21%) only when the object was the target of the experimenter’s action (OT neurons; Fig. 4 *C* and *D*), although the monkey neither moved nor prepared to move in these trials (Fig. 1 *E* and *F*), and the majority (*n* = 40, 48%) discharged during the presentation of the object both when it was targeted by the monkey’s and by the experimenter’s action (SOT neurons; Fig. 4 *E* and *F*). In this latter set of neurons, we could quantitatively assess the relationship between neural processing of object as a target for self-action (execution task) or another agent’s action (observation task).

**Fig. 5.**
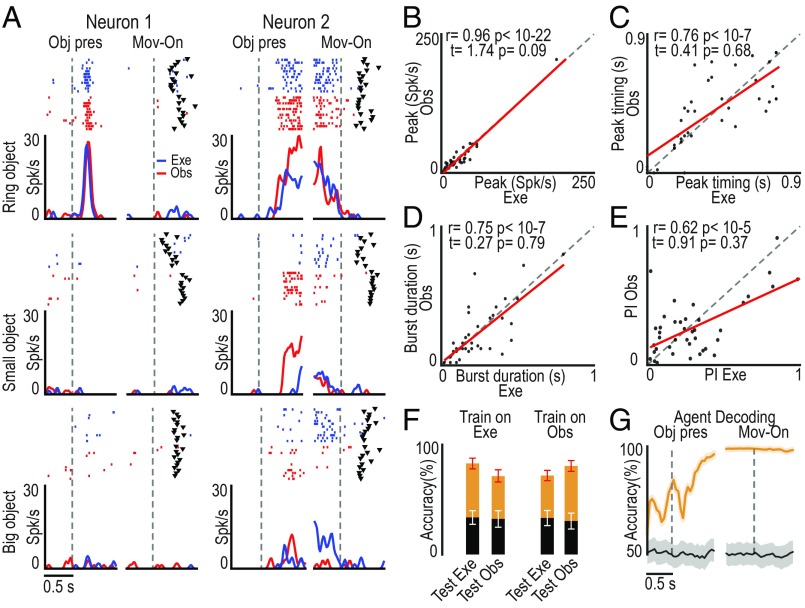
Relationship between the response of SOT object-related neurons (*n* = 40) to the visual presentation of the target for self (execution task) or other’s (observation task) action. (*A*) Single-neuron responses (raster and line plot) during task execution (Exe, blue) and observation (Obs, red) aligned (vertical dashed lines) on object presentation (Obj pres) and (after the gap) movement onset (Mov-on) with the three target objects. (*B*–*E*) Correlation plots calculated on the 0.8 s following object presentation in the execution and observation task of neurons peak of activity (*B*), peak activity timing (*C*), burst duration (*D*), and object PI (*E*) (*SI Appendix*, Fig. S4*B*). (*F*) Accuracy in the classification of the target during object presentation in execution and observation trials based on training a classifier with data collected during either one of the two tasks. (*G*) Time course of the agent decoding accuracy based on data collected during task execution and observation. All conventions are as in Fig. 3.

The object presentation response of two different SOT object-related neurons recorded during Go trials of the execution and observation tasks is shown in Fig. 5*A*. The discharge profile and object selectivity of neuron 1 are impressively similar when different agents (monkey or experimenter) had to grasp it. This pattern of activity may thus reflect an agent-independent coding of a pure object affordance (21). Neuron 2 exhibits a different behavior, being active in both tasks but showing a stronger modulation when the object was a target for the experimenter than for the monkey. This pattern of activity may thus correspond to an agent-based representation of the object. To quantitatively assess the overall relevance of these two different modes of object processing by SOT object-related neurons, we statistically analyzed parameters of their activity in the two contexts (execution and observation). We found that peaks of activity (Fig. 5*B*), their timing (Fig. 5*C*), bursts’ duration (Fig. 5*D*), and preferences for objects (Fig. 5*E*) were all highly correlated and not significantly different between task execution and observation, suggesting a remarkable degree of similarity between the overall activity pattern during the two conditions. Cross-modal decoding carried out on this set of neurons provided further support to the hypothesis that they may encode the object’s affordance both when it was targeted by self and by another’s action (Fig. 5*F*). Indeed, by training the classifier with input data collected during the execution and observation tasks, it could discriminate among objects equally well and largely above-chance level with both execution (Fig. 5*F*, Exe) and observation (Fig. 5*F*, Obs) data, suggesting that visual information on objects can be extracted with similar degree of accuracy regardless of the agent (self or other) to which it is addressed. To directly test possible agent-invariant coding of visually presented objects, we applied a decoding algorithm to test whether it could discriminate the agent (self or other) to which the target was addressed based on SOT object-related neuron activity (*Methods*). We found highly over-chance agent decoding accuracy (Fig. 5*G*), indicating that, despite the remarkable similarities between their representations of objects targeted by self or other agents, even SOT object-related neuron activity reflects agent selectivity.

### Object-Related Neuron Activity Is Strictly Constrained to the Monkey’s Peripersonal Space.

**Fig. 6.**
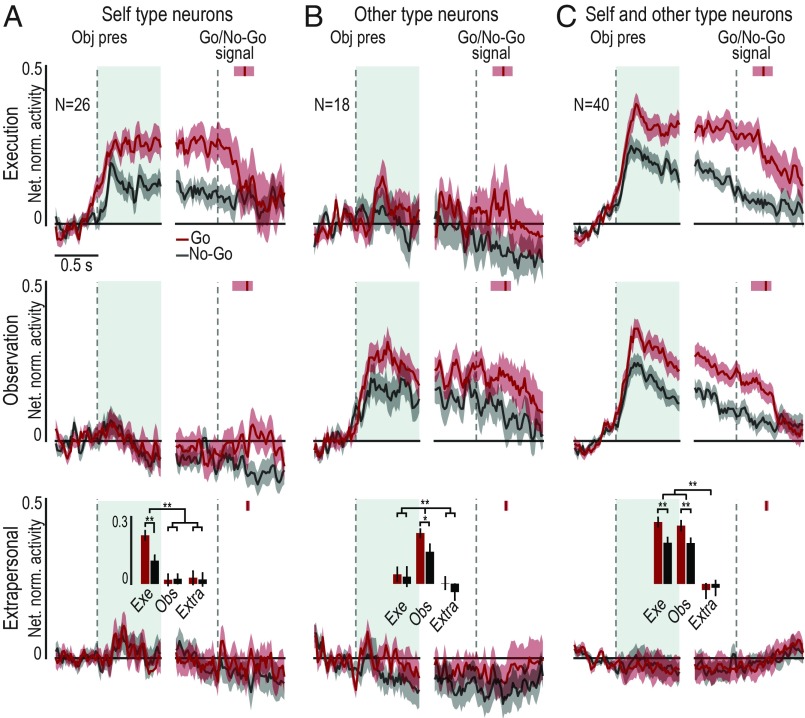
Space-constrained activity of object-related neurons. Population activity of ST (*A*), OT (*B*), and OST (*C*) neurons during task execution (*Top* row), task observation in the monkey’s peripersonal (*Middle* row), and extrapersonal (*Bottom* row) space during Go (red) and No-Go (gray) conditions. The activity is aligned (first dashed line on the *Left* of each panel) to object presentation (Obj pres), and then to the Go/No-Go signal (second dashed line on the *Right* of each panel). The response profile has been computed by averaging the response to the three different objects. The shading around each line indicates 1 SE. Histograms in the *Bottom* row represent the mean activity in each object presentation epoch (light-blue region following object presentation), compared among tasks with a 2 × 3 repeated-measures ANOVA (factors: Condition and Task) followed by Bonferroni post hoc test, *P < 0.05, **P < 0.01. Red vertical markers after Go/No-Go signal represent the median movement onset time, with indication of the 25th and 75th percentiles (shaded area around each marker).

To test whether “being at someone’s hand” (17) is sufficient to recruit area F6 object-related neurons, we compared neuronal responses to a visually presented object when it was a target (*i*) for the monkey’s own action, (*ii*) for the experimenter’s action performed in the monkey’s peripersonal space (Fig. 1*B*), and (*iii*) for the experimenter’s action performed in the monkey’s extrapersonal space (Fig. 1*D*). Regardless of the neuronal subpopulation (Fig. 6), object-related neurons do not discharge when the visually presented object is in the monkey’s extrapersonal space. This is also evident for SOT object-related neurons (Fig. 6*C*), indicating that objects are represented as a potential target for monkey’s own action (see *SI Appendix*, Fig. S5, for extrapersonal neuron responses).

Furthermore, object presentation responses are stronger during Go relative to No-Go condition in all of the three neuronal subpopulations. It is worth to note that in both subpopulations that encode the object when it is targeted by another agent (OT and SOT), the response reflects the same enhancement for the Go condition even during the observation task, when the experimenter, not the monkey, will grasp the object. This is particularly interesting for SOT object-related neurons, where we have already shown evidence of a close correspondence between the neuronal coding of object.

### Agent-Based Population Codes Dynamically Emerge from Object Presentation to Action Execution.

**Fig. 7.**
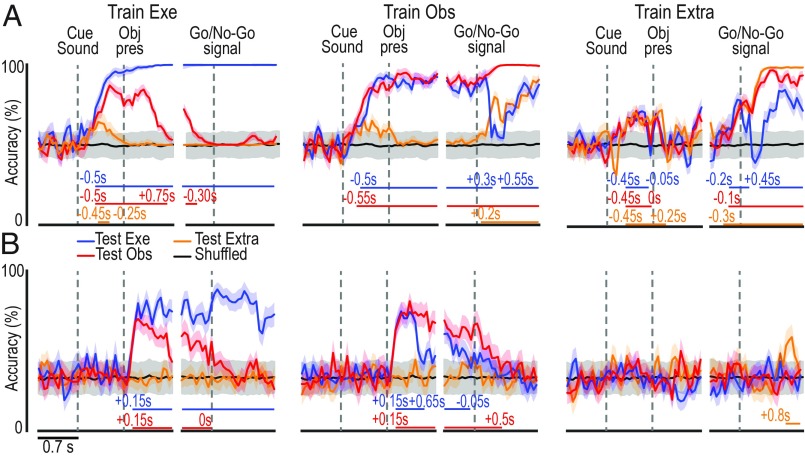
Cross-modal decoding of target object and Go/No-Go condition from area F6 population activity. (*A*) Classification accuracy over time of Go and No-Go trials in three different contexts, as a result of a maximum correlation coefficient classifier trained with a subset of data recorded in the execution (Exe), observation (Obs), or extrapersonal (Extra) task. The horizontal lines in the lower part of the plot indicate the time period during which the decoding accuracy was significantly and steadily above chance (black curves obtained with shuffled data) for at least 200 ms (*Methods*). (*B*) Classification accuracy over time of the type of object/grip type. Other conventions are as in *A*. Shaded areas around each curve indicate ±1 SD over resample runs. Results of the same analysis in individual animals are shown in *SI Appendix*, Figs. S6 and S7.

To assess the integrated contribution of F6 neurons to agent-based representations of objects and actions during task unfolding, we next applied cross-modal neural decoding methods to population data (20). All 306 neurons recorded from the two monkeys (111 neurons from M1 and 195 from M2) were included, with the unique selection criterion being well-isolated cells based on standard parameters (*Methods*). A classifier was first trained on a set of data collected in one task condition (execution, observation, and extrapersonal) to discriminate between Go and No-Go trials (Fig. 7*A*) and the type of object (Fig. 7*B*). Then the classifier’s decoding performance was tested on each condition to investigate whether, and to what extent, the population code generalizes across agents. The analyses were performed on data from each monkey, separately (*SI Appendix*, Figs. S6 and S7), but since the results were similar, here the data from the two animals have been combined.

After training the classifier on execution data, both Go/No-Go (Train Exe, Fig. 7*A*) and object (Train Exe, Fig. 7*B*) decoding accuracy was significant throughout the object presentation (premovement) period when tested with execution and observation, but not extrapersonal, data. A similar pattern was confirmed by training the classifier on observation data (Train Obs, Fig. 7) but not on extrapersonal data (Train Extra, Fig. 7), indicating that information conveyed by the cue sound and the target object can generalize between different agents, provided that they share the same operative space.

From the Go/No-Go signal onward, the classifier had to discriminate between performed and withheld actions (Fig. 7*A*) and grip types (Fig. 7*B*). Concerning action decoding (Fig. 7*A*, after the gaps), the classifier trained on execution data (Train Exe) could not decode other’s observed action in any other task contexts (Test Obs and Test Extra). In contrast, when trained on either observation (Train Obs) or extrapersonal data (Train Extra), it could reach significant decoding accuracy, although with clearcut differences, with both execution (Exe) and observation (Obs and Extra) data. This unidirectional visual-to-motor generalization of neural representation of actions suggests that somatomotor bodily signals determine agent specificity. Noteworthy, cross-modal decoding of grip type (Fig. 7*B*, after the gaps) did not show any evidence of generalization between the neural code associated with self- and other-action, suggesting that grip representation in area F6 completely depends on somatomotor rather than visual signal, and it cannot be achieved thorough the observation of other’s action. It is worth to note that when all object-related neurons are excluded from the population (*SI Appendix*, Fig. S8*A*), it is still possible to decode the visually presented object/grip type across tasks, but the decoding accuracy drop to chance level for most of the task unfolding period if only task-unrelated neurons are used (*SI Appendix*, Fig. S8*B*). Importantly, the decoding accuracy obtained with all neurons (Fig. 7*B* and *SI Appendix*, Fig. S8*C*, solid line) is greater than that obtained without object-selective neurons (*SI Appendix*, Fig. S8*C*, dashed line). Furthermore, object-decoding accuracy similar to that achieved with the entire dataset was obtained even with smaller subset of the data and by employing a different (Poisson naive Bayes) classifier (*SI Appendix*, Fig. S9). These findings suggest that, despite the crucial contribution of specific neuronal classes, the generalization of object representations across self and other is a computationally distributed function that does not critically depend on a selected population of neurons.

In summary, whereas area F6 appears to integrate auditory and visual information before movement onset to generate an agent-shared signal specifying “whether” and “how” self or other’s action will occur in the monkey’ peripersonal space, it then switches to a broader agent-based goal attainment signal during action unfolding.

## Discussion

In this study, we recorded neurons from the pre-supplementary motor area F6 (22), a crucial bridge between prefrontal and ventral premotor regions. This area has been proposed to play an important role in linking cognition to action (23), particularly in the social domain (11, 24). By employing a visuomotor task recently used to assess the neuronal responses of area F6 during object grasping (18), here we tested single-neuron activity from this area not only when monkeys performed the task but also when they observed an experimenter grasping the same objects within the same context. In addition to neurons providing agent-specific representation of self and/or others’ action, in line with previous studies (10, 14), our task allowed us to investigate object-related neuronal responses as well, and to demonstrate agent-specific processing of real objects in area F6.

As reported by previous single-neuron studies with arm-reaching tasks (10, 14), we found three main categories of action-related neurons: motor neurons becoming active during object grasping (ST neurons), visually responsive neurons discharging selectively during other’s observed action (OT neurons), and visuomotor neurons discharging during both self and other’s action (SOT neurons). This latter set of neurons behave as the classical “mirror neurons” reported in several nodes of the cortical grasping network (1), which provide a shared representation of self and other’s action (5, 6). Interestingly, whereas the motor response of area F6 mirror neurons allowed us to accurately decode the executed grip type, in line with recent findings (18), by applying cross-modal decoding methods we found no evidence that this code can generalize across the visual and motor domains, indicating the presence of an agent-specific coding of the grip type in this neuronal population and demonstrating that mirror neuron activity in area F6 can signal who (self or other) is acting. No previous study has ever directly investigated this issue in other brain areas, but the considerable differences between the visual and motor discharge often reported by previous studies in premotor (12, 25) and parietal (8) cortex suggest that this property may be widespread in cortical mirror neuron regions.

In contrast to premotor mirror neurons, which can often discharge differently depending on the observed grip type (7, 26–28), we found that this is not the case for F6 mirror neurons: Surprisingly, here we found that this information may be encoded by F6 object-related neurons, before any observed action onset.

As previously reported for action-related neurons, we found distinct sets of neurons encoding visually presented objects when they were targeted by the monkey’s own action (ST neurons), by the experimenter’s action (OT neurons), or both (SOT neurons). Self-type neurons likely represent the object as a potential target for the monkey, as previously hypothesized for visuomotor neurons in several other brain areas (29–32), including F6 (18). In line with this interpretation, they are characterized by stronger activity during Go relative to No-Go trials, robust and sustained visual-to-motor activity, and object-type selectivity. In contrast, OT neurons do not appear to represent the object features, but rather a broader predictive signal about another’s impending action, as previously hypothesized for some visuomotor neurons recorded from area F5 (12).

SOT object-related neurons are certainly the most intriguing class. In contrast with F6 mirror neurons, most of these cells and their overall population activity showed an impressive similarity in terms of temporal activation pattern and object selectivity during the execution and observation task. By applying cross-modal decoding methods to this neuronal population, in striking contrast with the results obtained with mirror neurons, we found robust evidence of a shared neural code underlying the representation of objects targeted by self and other’s action. At first glance, these findings may lead one to consider these neurons as the classical visuomotor object-related neurons, which respond to a visually presented graspable object in the same way every time the monkey is facing it (29, 33): In other terms, they may be simply representing the object’s affordances for the monkey, with no agent specificity. Against this idea, we showed that a classifier could robustly discriminate self- from other-trials based on SOT object-related neuron activity. Another important observation is that, during the observation task, Go and No-Go conditions are behaviorally identical from the monkey point of view. Nonetheless, the same differential response to the visually presented object between Go and No-Go trials in the execution task, which may reflect subsequent preparation for overtly performing the action, was also found in the observation task, where the monkey remained completely still in both conditions. Altogether, these findings suggest that object-triggered visuomotor responses in area F6 may allow observers to predict others’ impending action by recruiting the same motor representation they would activate if they were to act upon the same object in the same context.

On these bases, we propose that a mirroring mechanism exists in area F6 not only for actions but also for graspable objects. Compared with action mirroring, “object mirroring” appears to convey a richer and more precise information as to whether a reaching–grasping action will be taken, how it will be done, and who is about to perform it, enabling a subject to exploit part of the same neural circuit both to plan object-directed actions and to predict the actions of others long before any observable movement onset.

Predicting other’s action is usually possible even when the agent is located far from us, or on the virtual space of a screen (17, 34, 35), whereas the discharge of object-related neurons in area F6 appears to be strictly constrained to the observer’s peripersonal space. Because in the present study objects were presented from a subjective perspective in the peripersonal space and from a lateral perspective in the extrapersonal space, it may be argued that these viewpoint differences, rather than space, may account for the drop in neural selectivity for the object in the extrapersonal space. In contrast to this claim, our previous study on F6 neurons recorded from the same probes in the same animals clearly showed that neural selectivity for the object is abolished even in the peripersonal space if a transparent plastic barrier is interposed between monkey’s hand and the object (18). This finding demonstrates that “making far the near space” by keeping constant the viewpoint produces the same effect of presenting the object in the extrapersonal space, hence supporting the view that the peripersonal space is critical for F6 object-related neuron response. Future studies should investigate whether a similar neuronal mechanism with space-invariant features exists in other brain areas. Nonetheless, it seems reasonable to propose that the object-mirroring mechanism described here in area F6 does not simply play a general role in action anticipation, as previously described in the ventral premotor cortex (12, 15, 27), but it may allow a detailed, predictive representation of specific object-directed behaviors in a shared space for self and others’ impending action. From an evolutionary point of view, such a mechanism may also represent the precursor of the well-documented subjective view preference in the neural representation of observed actions (36–39), particularly when they occur in the observer’s peripersonal space (40). This subjective-view preference may be linked with own-hand visual feedback associated with overtly performed action, which is particularly relevant for the motor response of mirror relative to nonmirror neurons (39). These findings suggest that the space-constrained object-mirroring mechanisms here described may play a role in the predictive representation of other’s action within a context of social interaction.

To better understand the link between object- and action-mirroring mechanisms in area F6, we also employed cross-modal neural decoding methods to dynamically explore the possible generalization of the population code from self to other (and vice versa). Our results show that, following an early, mostly agent-shared representation of “whether” (Go/No-Go) and “how” (grip type) an object will be grasped, the population code progressively switches to a mostly agent-based goal attainment signal, devoid of any object/grip selectivity during other’s action observation. It is worth noting that the population code reflects information on the grip type only during action execution, indicating a strict dependency of this information from a somatosensory feedback or motor-related signal from other anatomically connected areas (41), which may further contribute to differentiate self and other’s action representation at the single-neuron level.

In conclusion, we provide evidence of an object-mirroring mechanism that allows recruiting the same motor representation of an object either for planning actions or to precisely predict how another agent will act when facing that object in the same context. Our findings support the idea that object-mirroring mechanisms play a more important role than action mirroring in simple and direct forms of context-based action prediction, with no need to recruit higher-order inferential processes.

## Methods

### Subjects and Surgery.

Experiments were carried out on two purpose-bred, socially housed macaque monkeys (M1, *Macaca nemestrina*, male, 9 kg, and M2, *Macaca mulatta*, male, 7 kg). Before recordings, monkeys were habituated to sit in a primate chair and to interact with the experimenters. They were then trained to perform the visuomotor tasks described below using the hand contralateral to the hemisphere to be recorded. When the training was completed, a head fixation system was implanted under general anesthesia (ketamine hydrochloride, 5 mg/kg, i.m., and medetomidine hydrochloride, 0.1 mg/kg, i.m.), followed by postsurgical pain medications (see ref. 42 for details). All experimental protocols complied with the European law on the humane care and use of laboratory animals (directives 86/609/EEC, 2003/65/CE, and 2010/63/EU), were authorized by the Italian Ministry of Health (D.M. 294/2012-C, 11/12/2012, and 48/2016-PR, 20/01/2016), and were approved by the Veterinarian Animal Care and Use Committee of the University of Parma (Prot. 78/12, 17/07/2012, and Prot. 91/OPBA/2015).

### Apparatus and Behavioral Paradigm.

Both monkeys were trained to perform, in different blocks, (*i*) a Go/No-Go execution task (Exe, Fig. 1*A*), (*ii*) an observation task (Obs, Fig. 1*B*), and (*iii*) an observation task carried out in the monkey extrapersonal space (Extra, Fig. 1*D*). We used a custom-made apparatus (*SI Appendix*, Fig. S1) allowing us to rapidly shift from one task condition to the other so that all tasks could be performed within the same session (see refs. 12, 39, and 40).

In the execution task, the monkey, sitting on a primate chair in front of the experimental setup (Fig. 1*A* and *SI Appendix*, Fig. S1), had to reach and grasp (Go conditions) or simply to fixate (No-Go conditions) three different objects: a ring, a small cone, and a big cone, each of which were to be grasped with a specific type of grip. Only one object was visible at a time, and all objects were presented in the same spatial position thanks to a sliding carousel. The animal had to grasp (or refrain from grasping) the object with the hand contralateral to the recorded hemisphere (left hand for M1, right hand for M2). All task conditions (Go/No-Go and types of object) were fully randomized within each task, and we collected 12 correctly performed trials for each condition. The temporal sequence of task events for the two conditions (Go/No-Go) of each task is the same used in a previous study (18) and described in Fig. 1*C*.

In the observation task (Fig. 1*B* and *SI Appendix*, Fig. S1), the apparatus, the target objects, and all of the task phases and conditions were as in the execution task. However, an experimenter was standing behind the monkey and performed the task with the same arm (left or right) used by the animal during the visuomotor task. The experimenter kept the hand on a small manipulandum, placed 10 cm next to the monkey’s hand: This contextual situation was readily associated by the monkeys to the observation condition (as shown by behavioral and EMG data; Fig. 1 *E* and *F*). In this condition, the monkey had only to remain still with its hand on the initial position and to maintain fixation during both Go and No-Go trials, observing the experimenter grasping or refraining from grasping the target.

As a control condition (Extra, Fig. 1*D* and *SI Appendix*, Fig. S1), in a third block of trials the experimenter performed the task in the monkey’s extrapersonal space (performing the task with the same hand used in the peripersonal space and by the monkey during the execution task), while the animal observed the scene from a lateral viewpoint (12). The apparatus, the target objects, and all task stages and conditions were the same as in the other tasks, but in this case the monkey could not reach any target, which was exclusively reachable by the experimenter. The task was presented in the space sector contralateral to the recorded hemisphere (left for M1 and right for M2).

The task phases were automatically controlled and monitored by LabView-based software, enabling the interruption of the trial if the monkey broke fixation, made an incorrect movement, or did not respect the temporal constraints described above. In all of these cases, no reward was delivered. After correct completion of a trial, the monkey was rewarded with the same amount of juice in all conditions.

### Neuronal Recordings.

Neuronal recordings were performed by means of chronically implanted arrays of linear silicon probes (18, 43). All probes were implanted vertically, ∼1 mm laterally to the mesial wall (*SI Appendix*, Fig. S2). Previous reports provide more details about the methodology of probe fabrication (44), assembly (43, 45), and implantation (46), as well as about the functional characterization of the investigated cortical sites with intracortical microstimulation (18).

Extracellularly recorded signal was amplified and sampled at 40 kHz with a 16-channel Omniplex recording system (Plexon). Because of the channel-count limit of the system, we recorded different sets of 16 channels in each session (one per day): No resampling of activity from the same channels in subsequent session was performed, and all single-neuron data included in this work have to be considered as completely independent, individual units. Spike sorting was performed on-line on all channels using dedicated software (Plexon), but all final quantitative analyses, including spike sorting, were performed off-line, as described in the subsequent sections.

### EMG Recordings.

EMG activity was recorded during separate sessions at the end of the single-neuron recording period. In both monkeys, we used couples of surface electrodes (Ag–AgCl) placed over a proximal [deltoid (DEL)] and a distal [extensor digitorum communis (EDC)] muscle of the arm contralateral to the hemisphere recorded during the electrophysiological experiments. Data were bandpass filtered between 30 and 500 Hz (fourth-order Butterworth), rectified, and averaged over trials. Statistical analyses were performed using the epochs of interest described below, and the same ANOVAs (followed by Bonferroni post hoc tests) applied to single-unit analysis (see below).

### Recording of Behavioral Events.

Distinct contact-sensitive devices (Crist Instruments) were used to detect when the agent (grounded), either the monkey or the experimenter, touched the metal surface of the starting position or one of the target objects with their hand. To signal the object pulling onset and holding phase, an additional device was connected to a switch located behind each object. Each of these devices provided a transistor–transistor logic (TTL) signal, which was used to monitor the monkey’s performance and to control the generation and presentation of the behavioral paradigm’s auditory and visual cue signals.

Eye position was monitored in parallel with neuronal activity with an eye-tracking system consisting of a 50-Hz CCD infrared sensitive video camera equipped with an infrared filter and two spots of infrared light. Analog signal related to horizontal and vertical eye position was fed to a computer equipped with dedicated software, enabling calibration and basic processing of eye position signals. The monkey was required to maintain its gaze on the fixation point (tolerance radius, 5°) throughout the task. The projection of the fixation point on the half mirror (*SI Appendix*, Fig. S1) allowed the monkey to see it even during object presentation (hence in full light) and object grasping. The eye position signal was monitored by the same LabView-based software dedicated to the control of the behavioral paradigm.

The same software also generated different digital output signals associated with auditory and visual stimuli, the target object presented in each trial, the reward delivery, and possible errors made by the monkey during the task (e.g., breaking fixation). All signals and TTL events generated during task unfolding were fed to the recording system to be stored together with the neuronal activity and subsequently used to construct the response histograms and the data files for statistical analysis.

### Definition of Epochs of Interest.

We focused on the following epochs of interest, defined in the same way for execution, observation, and extrapersonal tasks: (*i*) baseline, 0.5 s before object presentation; (*ii*) object presentation, from 0 to 0.5 s after switching on the light; (*iii*) premovement, 0.5 s before reaching onset (detachment of the hand from the starting position); (*iv*) reaching–grasping, from reaching onset to pulling onset (of variable duration, calculated on a trial-by-trial basis); (*v*) object holding, from pulling onset to 0.5 s after this event. Note that during baseline the monkey was still on the starting position, staring at the fixation point, and was already aware of whether the ongoing trial was a Go or a No-Go trial: this enabled us to assess possible variations in neural discharge linked specifically with the subsequent task stages within the current experimental context.

### Single-Unit Analysis.

The raw neuronal signals were high-pass filtered off-line (300 Hz). Single units were then isolated using principal component and template matching techniques provided by dedicated off-line sorting software (Plexon), and characterized with conventional criteria (46). Then, spike trains from different trials were compared using two distinct repeated-measures ANOVAs to test for possible responses to action and object. Both analyses were applied to each neuron response in the main execution and observation task, separately.

To test action-related responses, we considered neuronal activity during Go trials and applied 3 × 4 repeated-measures ANOVAs (factors, Object and Epoch), with the factor Epoch including baseline, premovement, reaching–grasping, and object-holding epochs defined above. To test object-related responses during Go trials, we applied a 3 × 2 repeated-measures ANOVA (factors, Object and Epoch), with the factor Epoch including baseline and object presentation epochs, as defined above. The significance criterion for ANOVAs was set to *P* < 0.05, followed by Bonferroni post hoc test (*P* < 0.01) in case of significant interaction effects. Neurons with a significant effect of the factor Epoch, either as a main or interaction effect, were considered as “task related” and classified as “action related”, “object related,” or “both,” depending on which analysis (or analyses) yielded the significant result (Fig. 1*G*). Object- and action-related neurons were further distinguished based on their agent selectivity: “self-type,” if they responded significantly only during the execution task; “other-type,” if they responded significantly only during the observation task; or “self- and other-type,” if they responded significantly during both tasks (Fig. 1*H*).

The same two types of repeated-measures ANOVAs were applied to each task-related neuron response in the extrapersonal task, as a control to verify whether task-related neurons could code object and/or other’s action in the monkey’s far space (*SI Appendix*, Fig. S5).

All analyses were carried out using Matlab 2015a and Statistica (StatSoft).

### Heat Maps Construction.

Heat maps (Figs. 2 *A*, *C*, and *E* and 4 *A*, *C*, and *E*) have been built to show the temporal activation profile of individual neurons in selected populations. Each line represents the activity of a single unit averaged across 36 trials with the three different objects (*n* = 12 for each object). The color code represents the net normalized activity, computed as follows: for each neuron, a mean baseline value across the 36 trials was computed (500 ms before object presentation), and then subtracted bin-by-bin for the entire task period. Activity was aligned to the object presentation and the movement onset. Suppressed responses were flipped according to single-unit analysis results. Finally, the net activity was normalized to the absolute maximum bin value (in each individual cell) across the conditions. All final plots were performed using a bin size of 100 ms and steps of 20 ms.

Because the activity was averaged across objects, we superimposed a black line on each heat map representing the total number of neurons showing object selectivity in each bin (referred to the same scale of the heat map). Object selectivity was assessed bin-by-bin with a one-way repeated-measures ANOVA (factor, object) with a significant criterion of *P* < 0.05 (uncorrected).

### Population Analyses.

Population plots were obtained from the mean (±1 SE) population activity, obtained bin-by-bin using as input the single neuron data calculated as described for the heat maps, with 60-ms bins slid forward in 20-ms steps.

Population data (Figs. 2 and 4) were statistically analyzed with 2 × 4 repeated-measures ANOVAs (factor, Task and Epoch), with a significance criterion of *P* < 0.05, followed by Bonferroni post hoc test (*P* < 0.01). Epochs 1 (baseline) and 3 (premovement) were the same used for single-neuron analysis, whereas epochs 2 (object presentation) and 4 (reaching–grasping) corresponded to 800 ms following each event (note that, in this way, epoch 4 always includes object pulling onset, which has a variable time lag across trials).

Additional population analyses were carried out to compare the object presentation response of ST, OT, and SOT neuronal subpopulation during Go and No-Go trials in all three different task contexts (execution, observation, and extrapersonal; Fig. 6). In this case, population data were analyzed by mean of 3 × 2 × 2 repeated-measures ANOVAs (factors: Task, Condition, and Epoch) applied separately to each neuronal subpopulation (*P* < 0.05, followed by Bonferroni post hoc test, *P* < 0.01).

### Correlation Analyses.

Correlation analyses were performed by means of a two-tailed Pearson’s correlation test (Matlab), carried out on different variables, namely: peak of activity, peak of activity timing, burst duration, and preference index associated with Go conditions of the execution and observation task. Each of these parameters was calculated, for each neuron, in a time window ranging from 0.5 s before movement onset to 0.8 s after this event in the case of action-related neurons (Fig. 3), and in a time window ranging from object presentation to 0.8 s after this event in the case of object-related neurons (Fig. 5). Each parameter, regardless of the reference time window, was calculated as follows.

#### Peak of activity.

A mean value across all 36 trials (averaging the three objects) was computed in 20-ms bins. The highest value (in spikes per second) within the reference time window was selected as peak of activity.

#### Peak of activity timing.

The time bins corresponded to the peak of activity relative to the reference event of interest (object presentation or movement onset, in the case of object-related and action-related neurons, respectively) were selected as peak of activity timing.

#### Burst duration.

We identified the first bin before (start) and after (end) the peak of activity corresponding to an activity value lower than 66% of the peak activity value: The time lag between start and end of the discharge period including the peak of activity was selected as burst duration.

#### Object preference index.

The preference index (PI) was calculated as defined by Moody and Zipser (47) with the following equation:

$$\left[\mathrm{PI}=\;\frac{n-\left(\frac{\sum r_{i}}{r_{\mathrm{pref}}}\right)}{n-1}\right],$$

where *n* is the number of objects used, *r*_*i*_ is the activity associated to each object, and *r*_pref_ is the activity associated with the preferred object. The index can range from 0 (lack of selectivity) to 1 (response to only one object).

To check for possible differences in the distribution of the values associated to each factor between the tasks, we also performed a paired-samples *t* test (*P* < 0.05). Furthermore, the distribution of PI indexes for each population, in each condition, was compared with 1,000 shuffled data distributions (*SI Appendix*, Fig. S4). For each repletion, we took real data across different objects (12 trials for each object) and we put them all together to get a set of 36 trials. Then, we mixed the object labels up, and based on the new (randomly assigned) labels we split the data again into three sets of 12 trials each. At the end, we compared the real data with the shuffled distribution with a paired *t* test.

### Decoding Analyses.

The methodology employed for the decoding analysis was the same as the one previously described by Meyers (20) and used in other studies (48–50). Specifically, we assessed the decoding accuracy of a maximum correlation coefficient classifier trained to discriminate between (*i*) Go and No-Go trials, (*ii*) the type of object used as target, and (*iii*) the agent (monkey or experimenter).

For each neuron, data were first converted from raster format into binned format. Specifically, we created binned data that contained the average firing rate in 150-ms bins sampled at 50-ms intervals for each trial (data point). We obtained a population of binned data characterized by a number of data points corresponding to the number of trials by conditions (i.e., 30 × 2 = 60 data points for Go/No-Go decoding; 10 × 3 = 30 data points for object decoding during Go trials; 30 × 2 = 60 data points for agent decoding during Go trials) in an *N*-dimensional space (where *N* is the total number of neurons considered for the analysis). Next, we randomly grouped all of the available data points into a number of splits corresponding to the number of data points per condition, with each split containing a “pseudopopulation,” that is, a population of neurons that were partially recorded separately but treated as if they were recorded simultaneously. Before sending the data to the classifier, they were normalized by means of *z*-score conversion so that neurons with higher levels of activity did not dominate the decoding procedure. Subsequently, the classifier was trained using all but one of the splits of the data and then tested on the remaining one: This procedure was repeated as many times as the number of splits (i.e., 30 in the case of Go/No-Go decoding, 10 in the case of object decoding; 30 in the case of agent decoding), leaving out a different test split each time. To increase the robustness of the results, the overall decoding procedure was run 50 times with different data in the training and test splits, and the decoding accuracy from all these runs was then averaged. The decoding results were based on the use of a maximum correlation-coefficient classifier. All of the analyses were performed on data collected from the two monkeys, as well as on the data collected from each animal, separately.

Note that when this procedure is applied by training the classifier to decode a specific variable (i.e., the type of object) in one condition (i.e., execution task) and testing its performance in another condition (i.e., observation and extrapersonal task), the results of this cross-modal decoding provide information on the generalization of the population code across conditions (Fig. 7 and *SI Appendix*, Figs. S6 and S7).

Cross-decoding analysis has been performed also by (*i*) focusing on a single 1,300-ms epoch (500 ms before and 800 ms after movement onset) of (self and other’s) action execution to test whether and to what extent the population code could generalize across agents, and (*ii*) focusing on a single 800-ms epoch of object presentation to self and other to test whether population code of object type could generalize across potential agents.

To assess whether the classification accuracy in the various analyses was above chance, we ran a permutation test using the Neural Decoding Toolbox in which the decoding analysis was run with the labels to be classified randomly shuffled, to obtain a null distribution to be compared with the accuracy of the decoding carried out on real data. The shuffling/decoding procedure was run 50 times. The *P* value was found by assessing, at each point in time, how many of the points in the null distribution were greater than those in the real decoding distribution and selecting only periods of at least three consecutive significant bins to visualize significant decoding accuracy in the plots (Fig. 7 and *SI Appendix*, Figs. S6–S8). The decoding results were only considered statistically significant if they were greater than all of the shuffled data tested in the null distribution.

## Supplementary Material

Supplementary File
