# Supplementary material for: Agent-based representations of objects and actions in the monkey pre-supplementary motor area
Source: Proc Natl Acad Sci U S A. 2019 Jan 29;116(7):2691–700. doi: 10.1073/pnas.1810890116 (PMC6377463; doi:10.1073/pnas.1810890116)
Supplement: Supplementary File [file pnas.1810890116.sapp.pdf]

PNAS
www.pnas.org

Supplementary Information for

Agent-based representations of objects and actions in the monkey pre-supplementary motor area

Alessandro Livi, Marco Lanzilotto, Monica Maranesi, Leonardo Fogassi, Giacomo Rizzolatti, Luca Bonini

Corresponding authors: Alessandro Livi, Luca Bonini, Giacomo Rizzolatti
Email: alessandro.livi2@gmail.com; luca.bonini@unipr.it; giacomo.rizzolatti@unipr.it

This PDF file includes:

Figs. S1 to S9

www.pnas.org/cgi/doi/10.1073/pnas.1810890116

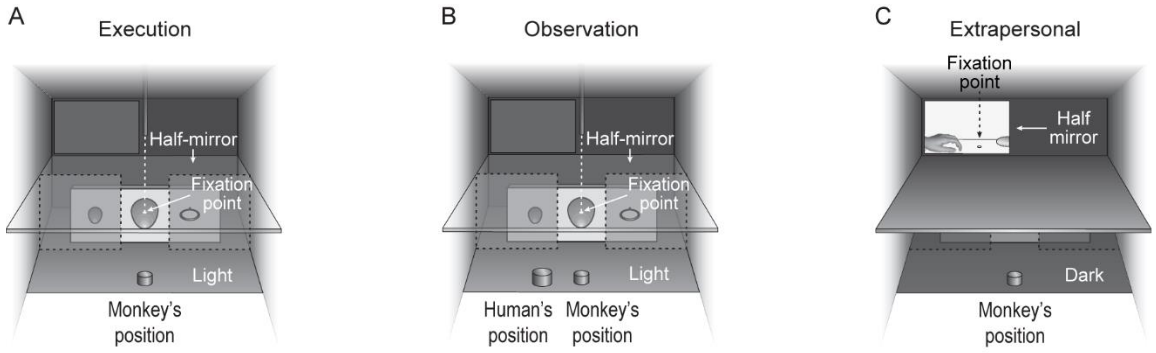

Fig. S1. Behavioral apparatus. Box and apparatus seen from the monkey's point of view settled for (A) the Execution task, (B) the Observation task and (C) the Extrapersonal task. The half-mirror allowed the animal to see the target only when the lower sector of the box was illuminated (object presentation). The fixation point was set to be in the center of each target. Human's position indicates an additional manipulandum placed 10 cm on the side of the monkey's hand during the Observation task. In the extrapersonal task, the half mirror allowed the animal to see (from a side-view) the object and subsequently the experimenter's action only when the light was turned on (object presentation). The fixation point was located in between the hand's starting position and the target object. The action was executed in the hemifield and with the hand contralateral to the recorded hemisphere.

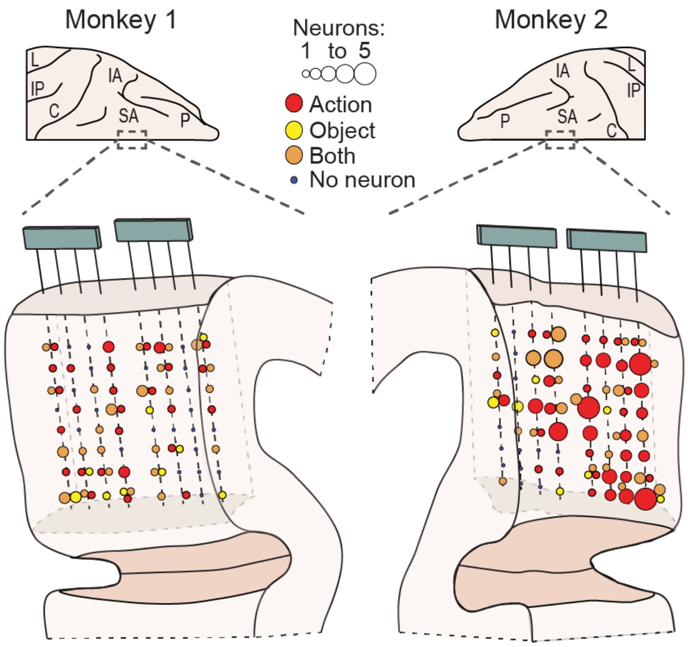

Fig. S2. Distribution and localization of Action- and Object-related neurons. Reconstruction of the anatomical distribution of task-related neurons in the investigated regions for Action (red), Object (yellow) and neurons responsive for both (orange). L, lateral sulcus; IP, intraparietal sulcus; C, central sulcus; IA, inferior arcuate sulcus; SA, superior arcuate sulcus; P, principal sulcus. Reconstruction of the recorded region was performed as described in a previous study (Lanzilotto et al, 2016) carried out with data recorded from the same animals and chronically implanted probes.

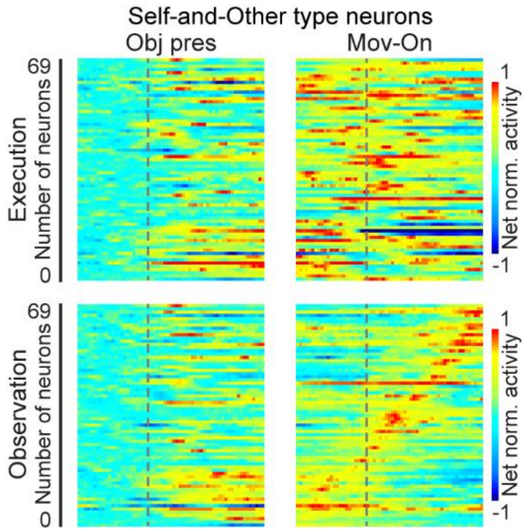

Fig. S3. Single neuron activity of SOT action-related neurons ordered based on the timing of their peak of activity during action observation. All conventions as in Figure 2E. The same neurons shown in Figure 2E here have been aligned based on their peak of activity timing during action observation. It emerges clearly the overall stronger activity during execution and the weak temporal relation between the activation pattern of these neurons in the two conditions (see also Figure 3 for quantitative measures).

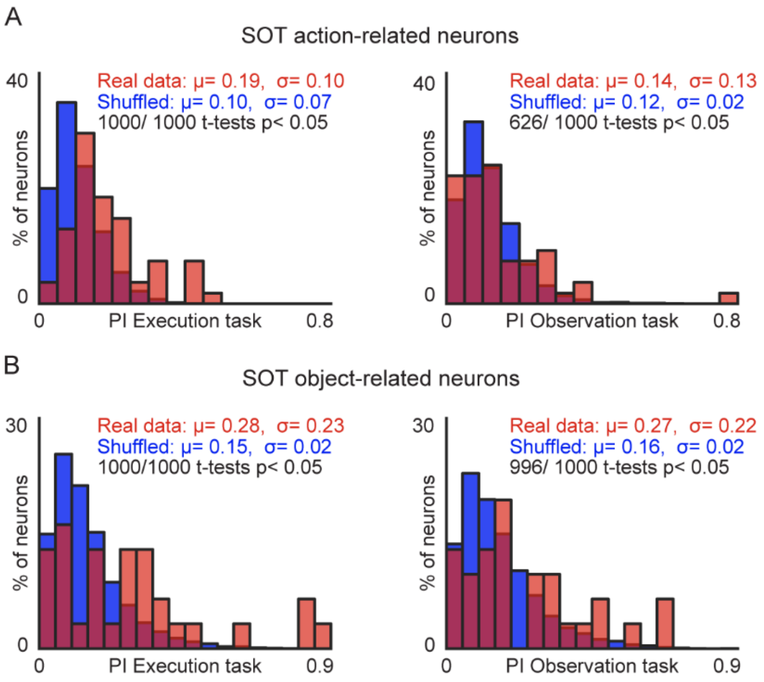

Fig. S4. Comparison between the distribution of Preference Index (PI) values calculated with real or shuffled data. To create shuffled data, the association of each trial to the object used (ring, small cone and big cone) was randomized for each SOT action-related (n=69) and object-related (n=40) neuron. PI was then calculated on 1000 repetitions of the shuffling procedure. The

distributions of PI values obtained from real (red) and shuffled (blu) data have been compared (t-test $p<0.05$) in action-related neurons (A, same data of Figure 3) and object-related neurons (B, same data of Figure 5) during both the execution and observation task. The results indicate that PIs calculated on real data are significantly ($p < 10^{-3}$) greater than those calculated on shuffled data whenever the neuronal population actually encodes object/grip type. Indeed, the only exception is represented by PI of action-related neurons tested during the Observation task (histogram in the upper right panel of the figure below): this population appears to be unable to encode the observed grip type even based on all the other analyses we performed (e.g. black line concerning grip selectivity in the lower panel of Figure 2E, Figure 3F and Figure 7B).

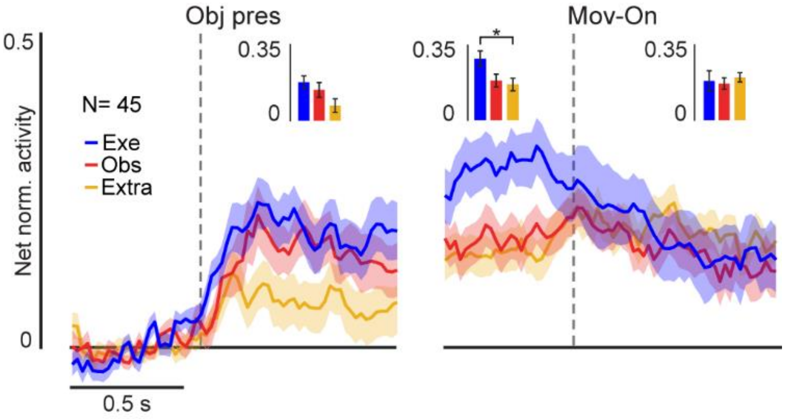

Fig. S5. Population responses of the 45 single units discharging significantly during the Extrapersonal task. All the recorded neurons (n = 306) were tested in the Extrapersonal task, and these units represent those significantly activated during this task. The population includes 29 neurons responding only to observed action, 6 responding to both object and action and 10 responding only during object presentation in the extrapersonal space. Time course and intensity of the mean net normalized population activity during the Execution, Observation and Extrapersonal tasks. The shading around each line indicates 1 standard error. Histograms in the insets of each panel represent the mean activity in each epoch for the three compared conditions. Repeated measure ANOVA 3x4 (Task, Epoch) revealed a significant interaction of the two factors [$F(6, 264) = 2.85$, $p= 0.01$]. *$p < 0.05$, Bonferroni post-hoc test.

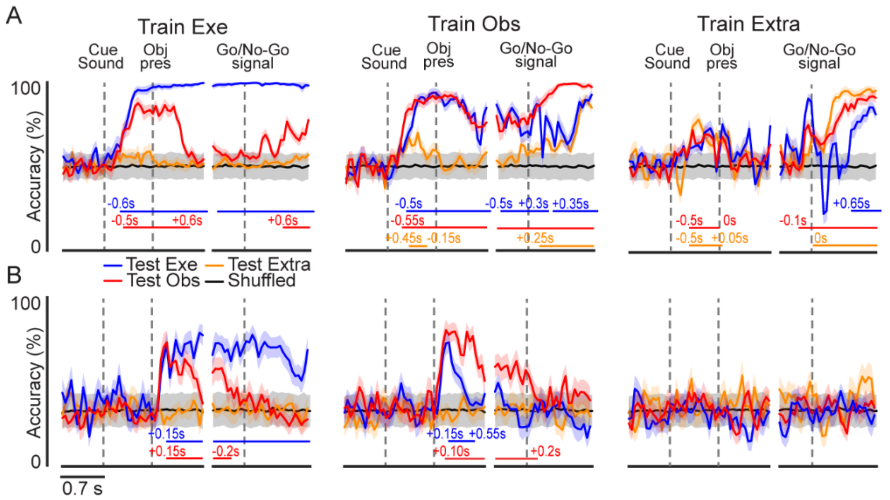

Fig. S6. Cross-modal decoding of target object and Go/No-Go condition from area F6 population activity of M1. (A) Classification accuracy over time of Go and No-Go trials in three different contexts, as a result of the classifier trained with a subset of data recorded in the execution (Exe), observation (Obs), or extrapersonal (Extra) task. (B) Classification accuracy over time of the type of object. The colored lines in the lower part of the plot show the time period during which the decoding accuracy was significantly and steadily above chance (grey curve obtained with shuffled data) for at least 200 ms (see Methods). Shaded areas around each decoding accuracy curve indicate standard deviation over resample runs. Other conventions as in Figure 7.

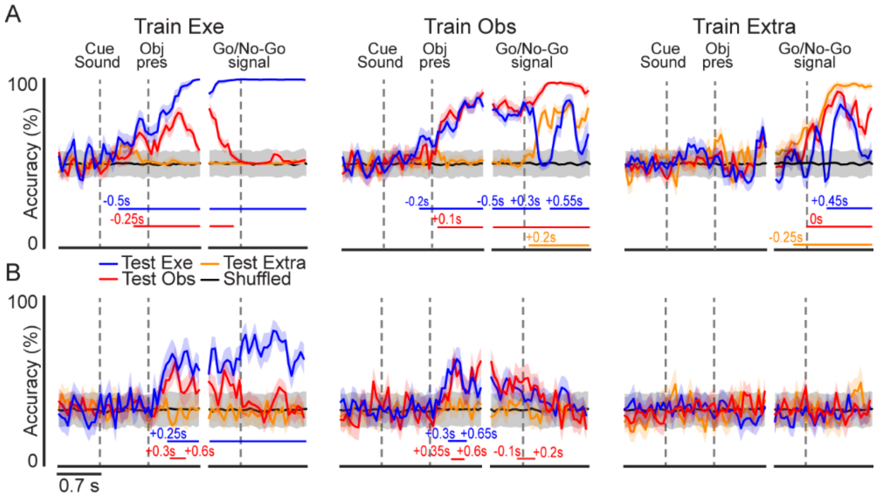

Fig. S7. Cross-modal decoding of target object and Go/No-Go condition from area F6 population activity of M2. (A) Classification accuracy over time of Go and No-Go trials and (B) of the type of object. All conventions as in Figure 7 and S6.

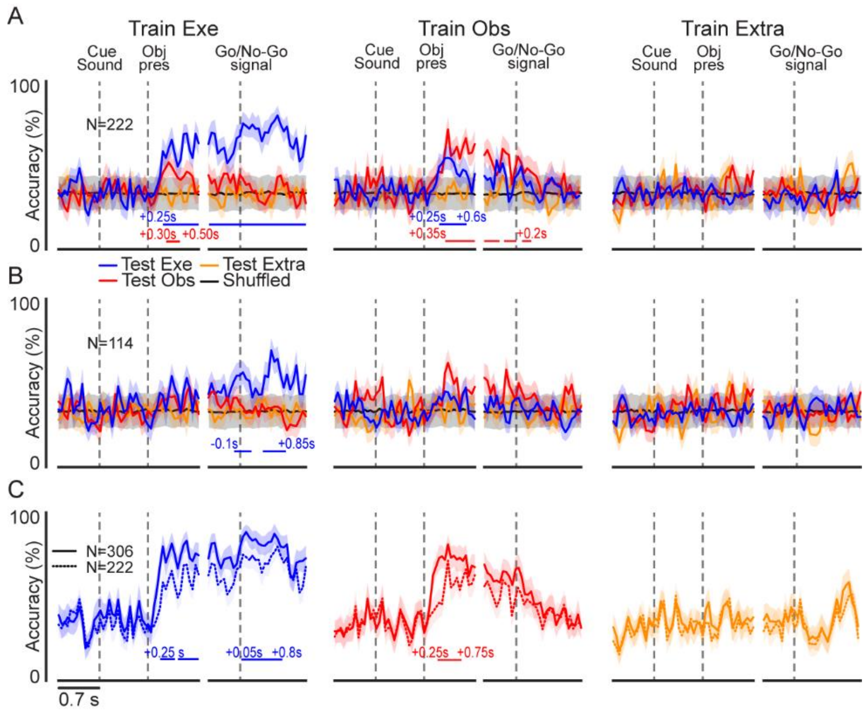

Fig. S8. Cross-modal decoding of target object from area F6 population activity after exclusion of different sets of neurons. (A) Classification accuracy without object-related neurons. (B) Classification accuracy without task-related neurons. Note that the classification accuracy above chance during the Execution task (blu line) is due to the presence, in this population, of neurons classified as task-unrelated that were modulated (though non-significantly) during action execution. (C) Comparison between classification accuracy obtained with all the recorded neurons (n=306, solid line as in Figure 7B) and that obtained from the population without object-related neurons (n=222, dashed line as in panel A). All other conventions as in Figure 7.

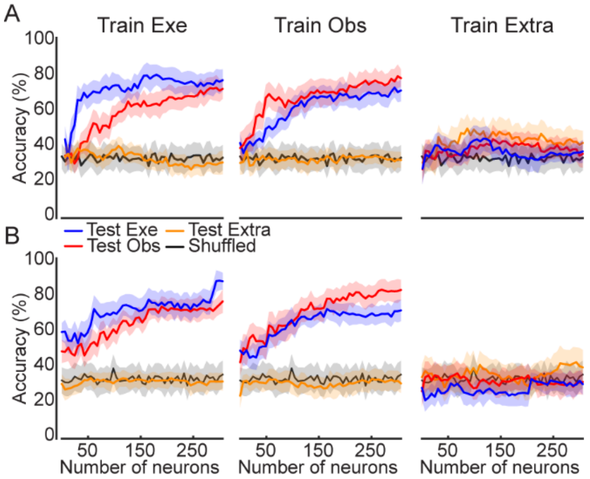

Fig. S9. Cross-modal object decoding performance as function of the sample size obtained with different classifiers. (A) Cross-modal decoding performance obtained with maximum correlation coefficient classifier applied to the entire data set. (B) Cross-modal decoding performance obtained with poisson naïve bayes classifier applied to the entire data set. Decoding

has been performed on the object presentation epoch, adding neurons in steps of 6 for a total of 306 neurons forming the entire pupulation. Curves show the mean (continuous curves) and standard deviation (shaded areas) over 50 iterations of random selections of each subset of neurons.
